# Hepatocyte UBE4A protects against MASLD and MASH by maintaining ER homeostasis via UBXN4 interaction

**DOI:** 10.64898/2026.10.07.757434

**Published:** 2026-10-13

**Authors:** Naga Lakshmi Challa, Vineet Babu, Molee Chakraborty, Matthew Hayes, Gaurav Kumar, Kyle S. McCommis, Craig W Vander Kooi, Danielle Carpenter, Isabela Ramos, Shivankitha Kattekola, Ameya Padakanti, Shikara Patel, Kaitlyn Mills, Sanchita Gupta, Louise Lantier, Brent A. Neuschwander-Tetri, Anutosh Chakraborty

## Abstract

Molecular understanding of the regulation of endoplasmic reticulum (ER) functions in healthy and MASLD livers is limited. Here, we elucidate that Ube4A, a cytosolic E3 ubiquitin ligase, protects against MASLD development by maintaining ER homeostasis during nutritional stress. Hepatocyte-Ube4A deletion (HUKO) augments Gubra-Amylin-diet (GAN-D)-induced steatosis, inflammation, insulin resistance, ER stress and markers of steatohepatitis of the liver in both sexes of mice. Proteomic studies reveal that in addition to altered metabolic and fibrogenic pathways, levels of many ER proteins are also changed in HUKO livers. Impaired ER functions sensitize HUKO livers to nutrition-or chemical (tunicamycin)-induced ER stress. Moreover, HUKO primary hepatocytes exhibit increased ER stress. Furthermore, Ube4A maintains ER homeostasis by facilitating ER-associated protein degradation (ERAD). The lipogenic protein SCD1 is identified as one of the ERAD targets of Ube4A. SCD1 stability and triglyceride accumulation are amplified in HUKO primary hepatocytes. The liver Ube4A-interactome studies identified the ERAD mediator UBXN4 as a major Ube4A interacting protein. Alphafold3-based molecular modelling and interaction site mapping analyses pinpointed the Ube4A binding residues for UBXN4. ER-localized Ube4A-UBXN4 complex facilitates ERAD (e.g., SCD1) and UBXN4-binding deficient Ube4A mutants fail to enhance this process. Finally, Ube4A and UBXN4 are downregulated in the livers of human MASLD and MASH patients and this complex is diminished in GAN-D-fed mouse livers. In conclusion, Ube4A preserves ER homeostasis by maintaining ERAD via its interaction with UBXN4, which maintains liver health. Enhancing levels and/or preserving interaction of these proteins may offer new therapeutic strategies for MASLD treatment.

